# The Immune Properties of Mesenchymal Stem Cells

**Published:** 2007-06

**Authors:** Marianne Castillo, Katherine Liu, Larrissa Bonilla, Pranela Rameshwar

**Affiliations:** 1 *Graduate School of Biomedical Sciences, UMDNJ-New Jersey Medical School, Newark, NJ, USA;*; 2 *Department of Medicine, UMDNJ-New Jersey Medical School, Newark, NJ, USA*

**Keywords:** mesenchymal stem cells, tissue repair, stem cells, bone marrow, interferon-gamma, graft vs host response, cytokine, cytotoxic cells

## Abstract

The goal of this review is to summarize current knowledge on the immune properties of mesenchymal stem cells (MSCs) and to discuss how these properties might affect clinical applications, in particular tissue regeneration. Mesenchymal Stem Cells (MSCs) are pluripotent cells with unique immune properties. They show immunoenhancing as well as immunosuppressive properties. It is the latter property that makes them stem cells of interest by scientists since they could be ideal for tissue regeneration, across allogeneic barrier. MSCs can transdifferentiate and differentiate into specialized cells. Although found mostly in the adult bone marrow, MSCs also reside in a variety of fetal tissues. In the adult bone marrow they act as “gatekeeper” cells regulating traffic in and out to the peripheral circulation and lymphatics. Their location within the vicinity of the bone marrow and periphery allows the MSCs, through their immune suppressor ability and antigen presenting property (APC) to maintain homeostasis in bone marrow function. There is potential for clinical therapy with MSCs. They have the potential to facilitate bone marrow transplantation by reducing graft-versus-host disease (GVHD). In addition, their immunosuppressive properties show promise for cell therapy across allogeneic barrier. Their role in the bone marrow, as it relates to hematological disorder is discussed.

## INTRODUCTION

**Figure 1:**
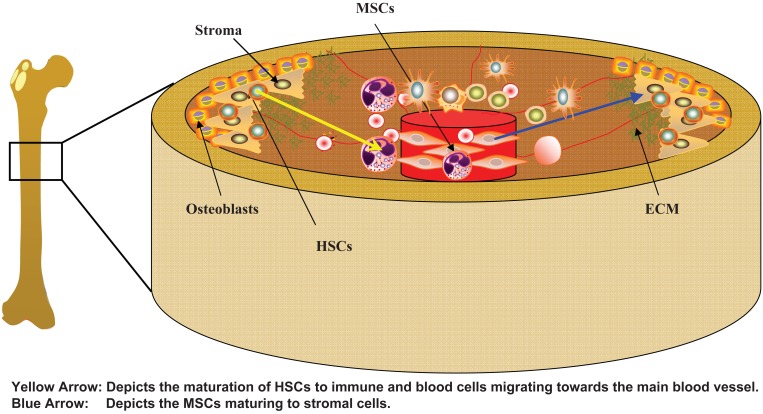
A cross-sectional view showing the relative cellular locations of the femur. Close to the endosteum shows hematopoietic stem cells interacting with bone marrow stroma. Mesenchymal stem cells are located close to the vasculature where they act as the “gatekeepers” of the bone marrow. HSC, Hematopoietic Stem Cells; MSCs, Mesenchymal Stem Cells; ECM, Extracellular matrix proteins.

Mesenchymal Stem Cells have the ability to develop into cells of the three different germ layers (1-4). They can be isolated from post natal and adult bone marrow (BM) (5), placenta (6), and various fetal tissues (7, 8). MSCs can be easily expanded by *in vitro* methods. However, it should be cautioned that it is still unclear how large numbers could be expanded for clinical application while maintaining efficiency. The type of surface markers and defined cell culture media are still under debate. In the adult BM, MSCs are one of the two resident stem cells, the other being the lymphohematopoeitic stem cell (HSCs). The latter are found mostly in the endosteal area of the BM where oxygen levels are low (9). In contrast, MSCs are found surrounding the blood vessels of the BM, specifically the central sinus, where oxygen levels are much higher (Figure 1) (10). As MSCs differentiate, their progenies migrate toward the endosteal area of the bone marrow to generate stromal cells, which support and maintain the functions of HSCs. The support leads to the differentiation into the major blood and immune cells of the body.

## OBJECTIVE

This review will discuss the versatility of MSCs with regards to their immune properties. The review will incorporate the relevance to tissue repair, transplantation, and also allude to the advantage with regard to ethical issues.

### Immune properties of MSCs

MSCs possess unique immune properties that make them leading candidates for regenerative medicine. Not only do they possess the ability to transdifferenitate into multiple organs; they also elicit immuno-suppressive effects that allow them to bypass allogeneic barriers (11). In the bone marrow, they are considered to be immune-gatekeeper cells, monitoring the cells entering and exiting the bone marrow. As gatekeepers, they can function both as antigen presenting cells (APCs) and immune suppressors, which is relevant in the maintenance of hematopoietic homeostasis within the bone marrow (12). Consistent with their APCs functions, the MSCs express Major Histocompatiblity Complex II (MHC-II), which provides them with the ability to present antigens to activated CD4+ T-Cells (12). Interferon gamma (IFN-γ) belong to a family of cytokines which are released in response to antigens. Therefore, as IFN-γ levels become elevated, they decrease the expression of MHC-II and revert to an immunosuppressive phenotype, preventing prolonged inflammation. These properties are relevant in the maintenance of homeostasis by protecting against infection and exacerbated inflammation which could alter hematopoietic functions.

MSCs also function in modulating many of the cells involved in eliciting immune responses. In mixed lymphocyte reactions (MLRs), they have been shown to suppress alloantigen and recall antigen induced lymphocyte proliferation (11-15). Although suppression was shown to be greatest when MSCs were added at the beginning of the MLR, inhibition was also seen when added later (16, 17). Additionally, MSCs seem to modulate other APCs, namely dendritic cells, by inhibiting the upregulation of facilitating molecules during their maturation, and also reduce the maturation efficiency (18-20). APCs isolated from co-cultures with MSCs showed reduced efficiency to mediate responses of CD4+ T-cells in mixed lymphocyte reactions (21). While the mechanism mediating the immunosuppressive effects of MSCs is unclear, studies have suggested suppression of immune responses may be mediated by soluble factors. This premise was derived from experiments in which MSCs and PBMCs, separated by a semi-permeable membrane showed no suppressive effects by the non-contact MSCs on PBMC proliferation (15, 16). In other studies, factors released from cultures of MSCs showed no evidence of immune suppression (11, 13, 22, 23). However, factors released from co-cultures of MSCs and lymphocytes showed immune suppression (13, 24), thereby suggesting a co-dependency for immunosuppression.

In general, cytotoxic responses, *in vivo*, are partly imparted by natural killer (NK) Cells and cytotoxic T-lymphocytes (CTLs). While CTLs target lysis of MHC-I mismatched cells, NK cells initiate a response against cells lacking MHC-I. NK cells produce interferon-γ (IFN-γ) when activated. In the presence of MSCs, IFN-γ production by NK cells is suppressed, indicating that the stem cells suppress the activation of NK cells (25). Additionally, MSCs lower the frequency of active CTLs in peripheral blood mononuclear cells (18). This effect is observed in a manner in which CTL response is indirectly proportional to the number of MSCs (18). The blunting of MSCs on CTLs is specific since fibroblasts derived from the same donor displayed no evidence of suppression (11). The fact that MSCs may suppress the activation of CTLs has vast significance in the treatment of graft-versus-host disease (GVHD). Although a mild form of GVHD may be desirable to avoid relapse of underlying disease, a severe form could be fatal to the patient (26, 27). Chung *et al.* (2004) demonstrated that co-transplantation of MSCs with hematopoietic cells resulted in both lowered IFN-γ levels and GVHD response. The ability of MSCs to lower the incidence of GVHD gives them vast potential in cellular therapy (28).

MSCs can also function as “veto cells” inducing tolerance by inhibiting lysis (11) and allogeneic lymphocyte proliferation by inducing the formation of CD8+ regulatory cells (24). Put together, all these immunomodulatory characteristics contribute to the extensive potential of MSCs in clinical therapies.

### Immunoregulatory role of MSCs in regeneration and therapy

In addition to tissue repair, MSCs can be utilized for the delivery of genes in various diseases. This property is possible mainly due to its immune suppressive functions. MSCs could respond to microenvironmental cues, but current research studies are aimed to utilize these stem cells for gene delivery as stem cells. In this type of clinical setting, it would be critical to maintain them as stem cells rather than differentiated cells since the former could act as immune suppressor. At this time, it is unclear if differentiated MSCs can maintain immune suppressor functions as compared to their more immature state as stem cells. The use of MSCs as cellular vehicle for gene delivery is clinically attainable since the cells could be prepared and delivered to patients ‘from the shelf’. The immune suppressive properties would allow allogeneic application and therefore prevent delays. This advantage is particularly useful in cases where delay could be fatal or debilitating such as in spinal cord injury.

Unlike hematopoietic stem cells, which are linked to controversies on their efficiency to generate cells other than blood and immune cells, MSCs are being accepted as adult stem cells with tissue regenerative potential (29-32). To reiterate tissue repair by most major stem cells is precluded by their allogeneic differences. The immune suppressive properties of MSCs make them likely to bypass the usual mechanisms of rejection (32, 33). While the veto property of MSCs is adequate for immune rejection, it might not be useful in organogenesis or in case where the MSCs form specialized tissue since at this time, the veto properties might be lost. In this regard, further studies are required to determine how the MHC Class I antigen, which would be different from the host, might prevent rejection. A useful point is that despite the immune suppressive properties of MSCs, they can nonetheless allow for immune response to common infectious agents, such as exposure to virus (34). Together, the experimental reports indicate that MSCs, if applied in a clinical setting, might show minimum toxicity. Aging disorder might be important to an understanding of the biology of MSCs since their dysfunctions have been reported for patients with aplastic anemia and myelodysplastic syndrome (35, 36). Pilot studies suggest that transplantation of MSCs in a patient with severe aplastic anemia showed clinical improvement (37).

### The response of MSCs during BM Insults

The BM is innervated (38), allowing a direct link to the central nervous system (CNS) via sympathetic nerve fibers (39, 40). This association between the BM and CNS indicates that any CNS effects may result in a hematopoietic response. Activation of the sympathetic nervous system in response to several normal, disease-related, or trauma-related stimuli is essential to maintain homeostasis in a constantly changing environment. This premise is supported by studies showing the hematopoietic changes by epileptic seizures and spinal cord injuries (36). In addition, studies in a murine model showed supplementation with noradrenaline led to improved survival after irradiation of bone marrow (36, 40). Other investigations have demonstrated disruption of MSCs is linked to hematological disorders (35, 42, 44).

Hematopoietic failure has been observed in experimental animals following shock and injury (43). In humans, BM insult has been observed in the red cell component and characterized by a persistent anemia, low reticulocyte counts and the need for repeated blood transfusions despite adequate iron stores (44-46). BM insult or failure is one facet of the multiple organ dysfunction syndrome and is commonly seen in patients recovering from severe trauma and hemorrhagic shock (T/HS) (47). During T/HS or infection, functions within the BM microenvironment could be altered, such as dysregulated cytokine production, consequently cause hematopoietic changes (48, 49). During injury there is a cascade of events mediated by cytokines and interleukins at intensified levels, such as inflammation and reparative signals (50-52). During this process cytokine production could be changes, release of bone marrow progenitors in the periphery and suppression of progenitor proliferation (53).

The question is what role do MSCs play in an otherwise healthy individual after infection, during trauma and hemorrhaging? Are MSCs homing towards regions of injury as a natural response to tissue repair or regeneration? Inflammatory mediators may stimulate MSCs to home or migrate to sites of injury, where cytokine levels such as stromal cell-derived factor 1alpha, tumor necrosis factor-alpha (TNF-α), and Interleukin 1 beta (IL-1β) are present. These proinflammatory cytokines also serve as signals for differentiation. Segers *et al.* (54) demonstrated that MSCs and cardiac microvascular endothelium have a biological and molecular basis for an intercellular interaction, and that both cell types can be activated for mutual interaction by proinflammatory cytokines such as TNF-1α and IL-1β. From their observations it has been hypothesized that circulating MSCs may serve as a source for regeneration of damaged myocardial cells. A downside to this response may be MSCs transdifferentiating into tissues not found in the area of injury, and the presence of such cells are likely to be harmful and may lead to further damage of neighboring organs.

During infection in the BM, the MSCs may respond to infection through their antigen presenting properties (12). Low-level infections may easily be regulated to prevent exacerbated inflammation in the BM, which would be harmful to hematopoiesis (12). However, in presence of an acute infection, the MSCs may not be able to cope with the clearance of the infectious agent. Further investigations are needed to further elaborate MSCs’ role in an acute infection response, where coping mechanisms may become attenuated leading to BM failure.

## CONCLUSION

The immunological abilities of MSCs can often function as the proverbial double edged sword. Investigations to harness the versatile enhancement and suppressing activities of MSCs will doubtlessly prove to be an increasingly vital area of study in the future. The ability of MSCs to differentiate into cells of different lineages, their modulation of the GVHD, their intimate interaction with neuronal factors results in immensely promising candidates for clinical therapies in regenerative medicine.
